# Die KomMent-Studie: ein Pilotprojekt zur strukturierten interprofessionellen Kommunikation in der Uroonkologie

**DOI:** 10.1007/s00120-022-01945-x

**Published:** 2022-10-07

**Authors:** Jana Jünger, Dominik Fugmann, Barbara Hinding, Ulrike Necknig, Stefan Bushuven, Stefanie Zschäbitz, Nancy Steiner, Peter Albers, Markus Giessing

**Affiliations:** 1 Institut für Kommunikations- und Prüfungsforschung gGmbH, Heidelberg, Deutschland; 2 Studiengangsleitung Master of Medical Education, Medizinische Fakultät, Universität Heidelberg, Heidelberg, Deutschland; 3 Klinisches Institut für Psychosomatische Medizin und Psychotherapie, Medizinische Fakultät und Universitätsklinikum Düsseldorf, Heinrich-Heine-Universität Düsseldorf, Moorenstr. 5, 40225 Düsseldorf, Deutschland; 4 Institut für medizinische und pharmazeutische Prüfungsfragen, Mainz, Deutschland; 5 Rotkreuzklinik Lindenberg, Lindenberg, Deutschland; 6 Institut für Krankenhaushygiene und Infektionsprävention, Gesundheitsverbund Landkreis Konstanz, Konstanz, Deutschland; 7 Abt. Medizinische Klinik VI, Universitätsklinikum Heidelberg, Nationales Centrum für Tumorerkrankungen, Heidelberg, Deutschland; 8 Klinik für Urologie, Medizinische Fakultät und Universitätsklinikum Düsseldorf, Heinrich-Heine-Universität Düsseldorf, Düsseldorf, Deutschland

**Keywords:** Kommunikationstraining, Ärztliche Weiterbildung, Nationaler Krebsplan, Onkologie, Kompetenzerwerb, Communication training, Postgraduate medical education, National Cancer Plan, Oncology, Skills acquisition

## Abstract

**Hintergrund:**

Kommunikation und interprofessionelle Zusammenarbeit mit krebskranken Patient:innen ist herausfordernd. Ein strukturiertes Kommunikationstraining ist bisher nicht in die ärztliche Weiterbildung integriert. Ziel der Studie war es, die Machbarkeit eines 80 Unterrichtseinheiten (UE) umfassenden interprofessionellen Kommunikationstrainings (IKT), wie im Nationalen Krebsplan empfohlen, an einer Klinik mit uroonkologischem Schwerpunkt zu prüfen.

**Methode:**

Eine Bedarfsanalyse wurde mittels Fokusgruppen und Einzelinterviews durchgeführt. Die Lernziele wurden mit (inter)nationalen Lernzielkatalogen abgestimmt. Das IKT wurde mittels des „six-step approach“ nach Kern und „design-based research“ erarbeitet. Die Inanspruchnahme und die Akzeptanz wurden evaluiert. Das IKT umfasste 6 Präsenzworkshops (50 UE) und eine Teamsupervision (10 UE). Für das individuelle arbeitsplatzbasierte Training (20 UE) wurden 6 definierte Settings identifiziert: Visite, Übergabe, Befundmitteilung, Aufnahme- und Entlassgespräch sowie ein Wunschsetting.

**Ergebnis:**

Die ärztliche Teilnahmequote an den Präsenzworkshops war 83,0 %, die pflegerische 58,3 %. Die Inanspruchnahme des arbeitsplatzbasierten Trainings lag bei 97 %. Die Ärzt:innen evaluierten das IKT sehr positiv (in Schulnoten Mittelwert [MW] 1,2 ± 0,4). Alle Teilnehmenden fühlten sich auf die Gespräche mit Patient:innen und Angehörigen besser vorbereitet. Zur Verstetigung wurden Ärzt:innen zu Mentoren ausgebildet.

**Schlussfolgerung:**

Die Implementierung eines IKT von 80 UE Umfang ist an einer urologischen Klinik erfolgreich durchführbar und führt u. a. durch eine Mentorenausbildung zu einer nachhaltigen Verbesserung der Kommunikationskultur.

## Abstract (en)

**Background:**

Communication and interprofessional collaboration with patients diagnosed with cancer is challenging. Structured communication training has not yet been integrated into postgraduate medical education. The aim of this study was to evaluate the feasibility of an 80-teaching unit interprofessional communication training (ICT), as recommended in the National Cancer Plan, at a clinic with a uro-oncological focus.

**Methods:**

A needs assessment was conducted using focus groups and individual interviews. Learning objectives were aligned with (inter)national learning objective catalogs. The ICT was developed using the six-step approach according to Kern and design-based research. Utilization and acceptance were evaluated. The ICT comprised six face-to-face workshops (50 teaching units) and team supervision sessions (10 teaching units). Six defined settings were identified for the individual workplace-based training (20 teaching units): Ward rounds, handover, reporting of medical findings, admission and discharge interviews, and a freely choosable setting.

**Results:**

Physician participation rates in the workshops were 83.0% and nursing participation rates were 58.3%. Utilization of the workplace-based training was 97%. The physicians evaluated the ICT very positively. All participants felt better prepared for discussions with patients and relatives. For continuity, physicians were trained as mentors.

**Conclusion:**

The implementation of an ICT with 80 teaching units is successfully feasible in a urological clinic and leads to a sustainable improvement of the communication culture, among other things through mentor training.

## Kernaussagen

1. Trotz des Bedarfs auf Seiten der Ärzt:innen gibt es bisher kein verpflichtendes, curriculär strukturiertes Kommunikationstraining in der ärztlichen Weiterbildung.
2. Ein interprofessionelles Kommunikationstraining mit 80 Unterrichtseinheiten (UE) Umfang entsprechend den Empfehlungen des Nationalen Krebsplans (NKP) ist an einer urologischen Klinik implementierbar.
3. Das vorliegende interprofessionelle Kommunikationstraining wurde von den Teilnehmenden sehr gut bewertet und zeigte eine sehr hohe Inanspruchnahme.
4. Besonders hohe Akzeptanz zeigte das individuelle arbeitsplatzbezogene Training (20 UE), bei dem reale Arzt-Patienten-Gespräche durch einen Mentor supervidiert werden.
5. Die Umsetzung des Konzepts und die nachhaltige Implementierung im eng getakteten Klinikalltag ist eine Führungsaufgabe.

## Einleitung

Eine gute Arzt-Patienten-Beziehung verbessert den Therapieerfolg und die Zufriedenheit der Betroffenen mit der Versorgung [1]. Bei Patienten mit lokalisiertem Prostatakarzinom konnte z. B. gezeigt werden, dass eine als gut wahrgenommene Kommunikation zu einer Verbesserung der Krankheitsbewältigung sowie der Lebensqualität führt [2], wohingegen Kommunikationsdefizite zu einer Beeinträchtigung des Behandlungserfolgs, der Arzt-Patient-Beziehung und der Lebensqualität beitragen [3, 4].

Die Behandlung onkologischer Patient:innen gestaltet sich oft herausfordernd: Starke Emotionen wie existentielle Ängste müssen empathisch im Behandlungsgespräch beachtet und professionell begleitet werden. In der Uroonkologie stellen sensible und schambesetzte Themen wie sexuelle Funktion und Kontinenz regelhaft eine zusätzliche kommunikative Herausforderung für die Behandelnden dar. Insbesondere Berufsanfänger:innen können so in Überforderungssituationen geraten [5].

Prostatakrebspatienten geben im Vergleich zu anderen Krebspatient:innen eine geringere psychosoziale Belastung an [6], jedoch ist die Suizidrate bei Patienten im lokalisierten Stadium 6fach erhöht [7]. Gute kommunikative Fertigkeiten könnten somit gerade bei dieser Patientengruppe von Vorteil sein, um eine bedarfsgerechte Versorgung zu gewährleisten.

Ein Kommunikationstraining kann nachweislich zu signifikanter Verbesserung kommunikativer Fertigkeiten führen [8, 9] und wird von uroonkologisch tätigen Ärzt:innen auch gewünscht [10]. Ärzt:innen wünschen sich eine Freistellung von der Arbeit für die Trainings [11]. Zwei systematische Übersichtsarbeiten zeigen, dass ein Training von mindestens 24 h effektiver ist als kürzere Trainings [8, 12]. Besonders die Integration in den klinischen Alltag wird von den Teilnehmenden (TN) als herausfordernd empfunden und als ein Implementierungshindernis beschrieben. Als eine Möglichkeit zur Verbesserung wird die individuelle Supervision am Arbeitsplatz empfohlen [10]. Weiterhin wird als Diskrepanz von den TN gesehen, dass das Gelernte nicht über alle Verantwortungsebenen der vorgelebten Kultur in der Klinik entspricht, weil auch in den höheren Hierarchieebenen häufig über die reine klinische Erfahrung hinaus kein formales Training über kommunikative Fertigkeiten oder Modelle erfolgt ist [13].

In den Empfehlungen des Nationalen Krebsplans (NKP) zur „Stärkung der Patientenorientierung“ im Ziel 12a und in der S3-Leitlinie Psychoonkologie werden eine Verbesserung der kommunikativen Kompetenzen bei den Gesundheitsberufen in Fort- und Weiterbildung gefordert [14, 15]. Auch wenn momentan in der ärztlichen Weiterbildungsordnung eine Schulung der kommunikativen Fähigkeiten prinzipiell vorgesehen ist [16], mangelt es an einem verbindlichen Curriculum.

In den Umsetzungsempfehlungen des NKP zum Ziel 12a wird ein Curriculum mit 80 UE für onkologisch tätige Ärzt:innen vorgeschlagen [17]. Zielsetzung der vorliegenden Studie sind die inhaltliche und didaktische Ausgestaltung eines strukturierten interprofesionellen Kommunikationstrainings (IKT) mit 80 UE und die Untersuchung seiner Umsetzbarkeit in der ärztlichen Weiterbildung am Beispiel einer urologischen Klinik. Als innovatives Element wurde sollte ein Teil des Trainings auf dem Arbeitsplatz währen der Routinearbeit umgesetzt werden.

## Material und Methoden

Das KomMent-Projekt („Förderung der *Kom*munikationskompetenz im Rahmen der ärztlichen Weiterbildung – Entwicklung und Implementierung eines *Ment*orings am Beispiel der urologischen Onkologie“) wurde durch das Bundesministerium für Gesundheit gefördert (Förderkennzeichen: ZMVI1-2517 FSB 020). Ein positives Ethikvotum wurde erteilt (Heinrich-Heine-Universität Düsseldorf, Nummer: 2018-221-ProspDEuA). Teilnehmende Ärzt:innen und Patient:innen wurden über das Projekt aufgeklärt und haben schriftlich eingewilligt.

Das IKT wurde am Universitätsklinikum Düsseldorf in der Klinik für Urologie erstmalig durchgeführt. Die Teilnahme war freiwillig.

Die Inanspruchnahme der 6 Präsenztrainings, der Teamsupervision und des individuellen arbeitsplatzbasierten Trainings wurde für jeden TN mittels Unterschriften protokolliert, die Akzeptanz wurde mittels semistrukturierten Interviews und Fragebögen evaluiert.

Insgesamt nahmen 8 Assistenzärzt:innen, 2 Oberärzte sowie 4 Pflegefachkräfte teil; 2 Assistenzärzt:innen verließen das IKT wegen eines Klinikwechsels, 1 Assistenzarzt kam während des laufenden Trainings hinzu.

Die Teilnahme von Vertretern der Leitungsebene wurde als strategisches Element in das Training integriert. Ziel war es zu gewährleisten, (1) dass die von den Assistenzärzt:innen gelernten Inhalte im klinischen Alltag umsetzbar sind, (2) dass die erarbeiteten Änderungen Einzug in die klinischen Strukturen finden und (3) dass das IKT nach Projektende durch „erfahrenere Mentoren“ verstetigt wird.

Für die Teilnahme am IKT erfolgte eine Freistellung von der Arbeit. Die Dienstpläne und OP-Pläne wurden im Rahmen des Design-based-research-Vorgehens im Vorfeld angepasst [18].

Für eine Bedarfsanalyse zur Ermittlung der inhaltlichen Schwerpunkte des IKT in der urologischen Abteilung wurden Gruppendiskussionen mit 7 Ärzt:innen sowie 6 Pflegefachkräften und Einzelinterviews mit 15 Pflegefachkräften und 6 Ärzt:innen durchgeführt. Die Interviews wurden transkribiert und mit MAXQDA 12 portable for Windows, VERBI-Software GmbH, Berlin, inhaltsanalytisch ausgewertet [19]. Zusätzlich floss eine Trainingsbedarfserhebung aus 6 deutschen urologischen Kliniken in die Auswahl geeigneter Lernziele mit ein [10].

Die Ergebnisse der Bedarfsanalyse wurden mit den Lernzielen für die studentische Ausbildung im nationalen kompetenzbasierten Lernzielkatalog Medizin (NKLM) und Gegenstandskatalog (GK; [20, 21]) sowie für die internationale onkologische Weiterbildung im Globalen Curriculum der American Society for Clinical Oncology (ASCO) und European Society for Medical Oncology (ESMO) abgestimmt [22].

**Figure 1:**
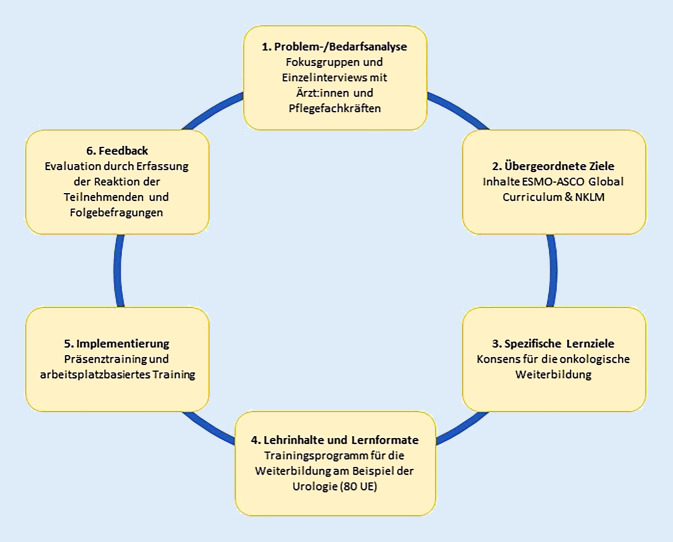

Die konkrete Ausgestaltung des IKT wurde entsprechend des „six-step approach“ der Curriculumsentwicklung nach Kern et al. ([23]; Abb. 1) mittels „design-based research“ und gemischt induktiv/deduktiv schrittweise erarbeitet [18]. Spezifische Problemfelder, die sich im Klinikalltag als Barrieren für die Integration des Gelernten darstellten, wurden im nächsten Training identifiziert und konkrete Lösungen entwickelt. Dies beinhaltete z. B. die Vorstellung der erarbeiteten Maßnahmen in der nächsten Leitungsrunde oder bei der Stationsübergabe, als auch deren Unterstützung durch die ärztliche und pflegerische Leitung.

## Ergebnisse

Von der ärztlichen und pflegerischen Leitung wurde der organisatorische und personelle Rahmen gesetzt, um Bedarfsanalyse, Konzeption und Implementierung des IKT parallel zum laufenden Klinikbetrieb zu ermöglichen. Alle Oberärzte wurden in die Planung einbezogen und die Freistellung der TN im Funktions- und Operationsplan gewährleistet.

### Bedarfsermittlung

Die Kommunikation hatte bisher einen geringen Stellenwert im klinischen Alltag. Die TN gaben an, aufgrund eng getakteter Termine und hohen Patientenaufkommens zu wenig Zeit zu haben, Patientengespräche angemessen zu führen. Häufige kommunikative Herausforderungen waren das Überbringen schlechter Nachrichten, unvorbereitete Gesprächssituationen und fehlendes Fachwissen, unerwartete Patientenreaktionen, Gespräche mit Angehörigen sowie der Umgang mit sensiblen Themen, kulturellen Unterschieden und Sprachbarrieren.

Für die interprofessionelle Zusammenarbeit wurde der Wunsch nach „besserer Kommunikation“, respektvollerem Umgang sowie nach mehr konstruktivem Feedback im Team geäußert. Der Informationsfluss bei den interprofessionellen Übergaben sowie die Einbindung der Pflegefachkräfte bei der Visite erwiesen sich als unzureichend. Übergeordnet kristallisierten sich die Themen Patientensicherheit, Infektionsprävention, Umgang mit Fehlern und verbesserungsfähige organisatorische Abläufe mit erhöhtem Kommunikationsaufwand heraus. Die Ergebnisse der Gruppeninterviews wurden in 12 Paraphrasen verdichtet und in einer Gruppendiskussion priorisiert (Tab. 1). Das am häufigsten genannte Lernziel war Kommunikation zwischen Ärzt:innen und Pflegenden.

**Table 1:** 

Identifizierte Themenfelder zur Bearbeitung	Punkte
Kommunikation zwischen Pflegenden und Ärzt:innen	7
Umgang mit der Emotionalität Anderer	6
Fordernde Patient:innen und Angehörige	5
Fehler- und Problemkommunikation	5
Visite	3
Übergabe	3
Kommunikation bei Mangel an Fachwissen oder Erfahrung	2
Feedback (geben und annehmen)	2
Dem Gesprächsanlass angemessene Räumlichkeiten	2
Umgang mit eigenen Emotionen	1
Standards und Leitlinien zur Unterstützung im Alltag	0
Umgang mit Unterbrechungen/Verfügbarkeit	0

Für das konkrete *arbeitsplatzbasierte Training* kommunikativer Fähigkeiten wurden in der Gruppenarbeit von den TN 6 klinische Alltagssituationen identifiziert: Visite, Übergabe, Befundbesprechung, Aufnahme- und Entlassgespräch sowie ein Wunschsetting.

### Struktur und Inhalt

Das IKT beinhaltete in 80 UE: Gruppen‑/Präsenztraining (50 UE), arbeitsplatzbasiertes Training (20 UE) und Teamsupervision (10 UE). Aufgrund der Bedarfsanalyse wurde das komplette Präsenztraining interprofessionell durchgeführt anstatt, wie ursprünglich geplant, nur in Teilbereichen.

Das *Gruppen‑/Präsenztraining mit 50* *UE* erstreckte sich über ein Jahr mit sechs Veranstaltungen im Abstand von 6 bis 8 Wochen zu jeweils 8–10 UE. Allgemeine Kommunikationsmodelle wurden interprofessionell erarbeitet und mit standardisierten Patient:innen (SP), also Schauspieler:innen, geübt. Spezifische Kommunikationsmodelle wurden mit SP in komplexen Szenarien erlernt (Tab. 2). Hierfür wurden ausschließlich uroonkologische Fallvignetten entwickelt.

**Table 2:** 

Thema		Inhalt und Methoden	Zeitlicher Umfang (UE)
1	Grundlagen	WWSZ-Modell: **w**arten, **w**iederholen, **s**piegeln, **z**usammenfassen. Buchmetapher	4
2	Umgang mit Emotionen	NURSE-Modell: „**n**aming, **u**nderstanding, **r**especting, **s**upporting, **e**xploring“. Rollenspiel	6
3	Überbringen schlechter Nachrichten	SPIKES-Modell: „**s**etting, **p**erception, **i**nvitation, **k**nowledge, **e**motion, **s**ummary“. Übung mit SP	6
4	„Prognostic awareness“	Prognostic-awareness-Modell. Briefing und Debriefing. Übung mit SP	6
5	Partizipative Entscheidungsfindung	Prozessschritte zur Umsetzung von partizipativer Entscheidungsfindung. Übung mit SP	6
6	Kommunikation mit Angehörigen	Kultursensible Betreuung und Umgang mit Angehörigen	6
7	Kulturelle und sprachliche Barrieren	Übung mit SP	
8	Sicherheitskultur	SHARP-Modell: „**s**et learning objective; **h**ear, what has been accomplished; **a**dress concerns; **r**eassure; **p**lan“. CUS-Modell: „I am **c**oncerned, I feel **u**ncomfortable, this is a **s**afety issue“. ECO-Modell: „**e**xplore, **c**larify, **o**utcome“. Übung mit SP: feedback, „speaking up“, Nachbesprechung einer kritischen Situation	6
9	Übergabe und Risikomanagement	SBAR-Modell: „**s**ituation, **b**ackground, **a**ssessment, **r**ecommendation“. SOAP-Modell: „**s**ubjective, **o**bjective, **a**ssessment, **p**lan“. Übung zur Prozessanalyse am Beispiel einer Übergabe. Übung mit SP: Überbringen der Nachricht nach einem Fehler bei der interprofessionellen Übergabe, kritischer Schaden eines Patienten nach mangelhaftem präoperativem Assessment	6
10	Sensible Themen	Kommunikation bei schambehafteten Themen. Übung mit SP	4

*SP* standardisierterte Patient:innen (Schauspieler:innen), *UE* Unterrichtseinheiten

Im *arbeitsplatzbasierten Training (20* *UE)* wandten die TN das Geübte unter Supervision eines Kommunikationstrainers in echten Patientengesprächen an, im Anschluss erfolgte ein videogestütztes Feedback.

Bei der intermittierenden *Teamsupervision (10* *UE)* stand die interprofessionelle Zusammenarbeit und Kommunikation im Mittelpunkt. Inhaltliche Schwerpunkte waren dabei Feedback, gewaltfreie Kommunikation, Sicherheitskultur, Problemlösungskompetenz sowie Konfliktmanagement.

### Teilnahme und Bewertung

Die ärztliche Teilnahmequote an den Präsenztrainings war 83,0 %, die pflegerische 58,3 % Die Inanspruchnahme des arbeitsplatzbasierten Trainings lag bei 97 %, ausgenommen zwei Ärzt:innen, welche das Training wegen eines Klinikwechsels vorzeitig beendeten (Tab. 3).

**Table 3:** 

Setting	TN 1 (UE)	TN 2 (UE)	TN 3 (UE)	TN 4 (UE)	TN 5 (UE)	TN 6 (UE)	TN 7 (UE)
Aufnahmegespräch	1	4	4	4	4	4	6
Visite	2	4	4	4	2	3	3
Übergabe	–	–	3	3	3	3	3
Befundbesprechung	1	1	3	7	8	6	5
Entlassgespräch	–	–	3	2	3	4	3
Sonstige	–	–	–	–	–	–	–
Insgesamt	4^a^	9^a^	17	20	20	20	20

*UE* Unterrichtseinheiten, *TN* Teilnehmende

^a^Abbruch des Trainings wegen Klinikwechsel

Die Ärzt:innen evaluierten das IKT sehr positiv (in Schulnoten MW 1,2 ± 0,4).

Durch das IKT fühlten sich alle TN auf die Gespräche mit Patient:innen und Angehörigen besser vorbereitet (Ärzt:in, DDBI, Welle 2, 40: „… ich fühl mich sicherer bei schweren Situationen (…) Da gibt’s nicht mehr so häufig so dieses, dieses völlige Ohnmachtsgefühl, Unwissenheitsgefühl oder so etwas, wie ich es vielleicht früher mal hatte.“). Dabei fanden die TN die Übungen mit den SP sehr hilfreich (Ärzt:in, EBTG, 3. Welle, 31: „Da fand ich dieses mit den Schauspielpatienten, so unangenehm es auch manchmal ist, wenn man es selber macht, hat mir irgendwie ein bisschen mehr gebracht – also, wenn man es irgendwie aktiv angewandt hat – als das immer nur so durchzusprechen.“) Die erlernten Kommunikationsmodelle konnten in den Arbeitsalltag integriert werden (Ärzt:in, EEHA, 3. Welle, 32: „… alleine dieses Handwerkszeug, verschiedene Tools, SPIKES, WWSZ, „prognostic awareness“, dass man die wirklich, wirklich einsetzt. Und die kannte ich einfach vorher nicht. Also das hat im Gespräch mit den Patienten viel gebracht.“ Ärzt:in, MTME Welle 2, 18: „Ich hatte heute auch einen, da hab ich aktiv nach Emotionen gefragt. Das hatte sehr gut geklappt.“). Als besonders positiv wurde die Bildung einer „Community of Practice“ erlebt, in der individuelle und gemeinsame Ziele durch die Gruppe unterstützt und eine respektvolle Feedbackkultur praktiziert werden (Ärzt:in, Freitextantwort im Fragebogen zur Evaluation der Präsenztermine: „Am Training hat mir besonders gut gefallen, dass es eine offene Diskussionskultur gab.“).

Von den TN der Pflege wurde bemängelt, dass das Training zu sehr auf ärztliche Bedürfnisse ausgerichtet war (Pflegefachkraft, FJDP, 2. Welle, 104: „Es wird dann mal versucht, dass wir einfach dazu gehören. Aber oft waren wir einfach nur Rahmenpersonen …“. Pflegefachkraft, FJDP, 2. Welle, 102: „… es waren meistens immer so, dass man so 80 zu 20 immer so’n bisschen – 80 eher medizinisch-ärztlicherseits und ein bisschen Kommunikation, das auch die Pflege mitbetrifft.“).

### Strukturelle Änderungen in der Klinik

Erkenntnisse aus diesem IKT führten im Umfeld der Arbeit zu neuem Verhalten (Pflegende:r, ZNLN, 2. Welle, 117: „… es hat sich schon einiges verbessert. Zum Beispiel jetzt haben die Ärzte bisschen mehr Zeit für Patienten.“ Ärzt:in, RRMI 3. Welle, 33: „Ich glaube es hat sich was verändert bei den Leuten, die mit in der Kommunikationsausbildung waren. Definitiv. Einfach, dass die offener sind, dass die zugehen auf die Leute, dass die einfach auch sehr gute Patientengespräche führen, also sie machen es wirklich exzellent, muss man wirklich sagen“). Es wurde festgelegt, bei Patientengesprächen den Funk abzugeben, und ein realistischer zeitlicher Umfang für Patientengespräche wurde abgeleitet (z. B. 30 min für schwierige Befundmitteilungen). Gesprächssituationen, die in einem separaten Raum stattfinden sollten, wurden definiert (z. B. Befundmitteilung, Behandlungsübergang von kurativ zu palliativ). Eine tägliche interprofessionelle Kurvenvisite wurde neu eingeführt (Ärzt:in, EEHA 3. Welle, 46: „… dass wir halt versuchen enger mit der Pflege zusammenzuarbeiten. Also gemeinsame Visiten, gemeinsame Übergaben, gemeinsame Tumorboards. Einfach, damit man, ähm, ja, mehr Wissen teilt, mehr Infos mitbekommt, ne. Und das hat ehrlich gesagt gut funktioniert …“).

### Nachhaltigkeit

Erfahrene Assistenzärzt:innen und Oberärzte wurden zu Mentoren ausgebildet, diesen wurden alle Assistenzärzt:innen der Klinik in Kleingruppen zugeordnet, die sich auch nach Abschluss des KomMent-Projekts weiterhin alle 6 Wochen treffen.

## Diskussion

Ein IKT mit 80 UE ist an einer urologischen Klinik in einem Zeitraum von 24 Monaten implementierbar.

Die Lernziele wurden aus einer Bedarfsanalyse und aus übergeordneten Curricula abgeleitet. Wie in üblichen Kommunikationstrainings [24–27], die jedoch mit meist 10–20 UE deutlich kürzer sind, wurden Themen wie Überbringen schlechter Nachrichten und Umgang mit Emotionen aufgenommen. Zusätzlich wurden jedoch z. B. „prognostic awareness“, (interprofessionelle) Übergabe sowie Sicherheits- und Fehlerkultur, die in der Bedarfsanalyse und internationalen Literatur als bedeutsam identifiziert wurden, in das IKT aufgenommen. Dadurch ergab sich ein Gesamttrainingsumfang von 80 UE, der damit scheinbar dem häufig genannten Wunsch nach einem ein- bis zweitägigen Training entgegensteht [11]. Es war zu vermuten, dass die Ärzt:innen antizipieren, dass die Integration längerer Trainings in den Arbeitsalltag nicht möglich sei: Entsprechend war die am häufigsten genannte Barriere gegen eine Teilnahme an einem Kommunikationstraining Mangel an Zeit. Konsequenz war, dass viele der Themen, bei denen ein Trainingsbedarf besteht [10, 20–22], in üblichen Trainings deshalb im Gegensatz zu unserem IKT nicht abgebildet wurden.

Analog dazu wurden als Hemmnis für gute Kommunikation in den Fokusgruppen und Interviews häufig Störungen des Settings durch Funk, Telefon oder anderes Personal genannt. Auffälligerweise wurden bei der Priorisierung gewünschter Inhalte für das IKT durch die TN diese Punkte jedoch nicht aufgenommen, weil die TN bezweifelten, dass ein IKT die generellen klinischen Abläufe positiv beeinflussen könnte (s. Tab. 1). Trotz der negativen Erwartungshaltung der TN im Hinblick auf Veränderbarkeit klinischer Strukturen wurden diese durch die Zusammenarbeit von Klinikleitung und IKT-Leitung verbessert: So wurden schwierige Gespräche nunmehr mit den Kolleg:innen abgestimmt, der Funk wurde für diesen Zeitraum abgegeben und es wurde auf Störungsfreiheit geachtet. Für die Konzeption von Trainings bedeutete dies einerseits TN-Wünsche zu berücksichtigen, aber auf der anderen Seite sinnvolle übergeordnete Ziele trotz negativer TN-Erwartung anzustreben.

Um bei der Implementierung des IKT das Dilemma zwischen fehlender Zeit im klinischen Alltag und hohem Trainingsbedarf zu lösen, wurden klinische Entscheidungsträger:innen in jeder Phase des Projekts konsequent eingebunden. Die klar unterstützende Haltung der Klinikleitung wurde deutlich durch die Anerkennung des IKT als Arbeitszeit sowie der Berücksichtigung bei der Ambulanz- und OP-Planung, als auch der Weiterentwicklung bestehender Organisationsabläufe aufgrund der Erkenntnisse aus dem IKT. Anfängliche Widerstände und Vorbehalte nicht teilnehmender Klinikmitarbeiter:innen hinsichtlich der Machbarkeit insbesondere im Hinblick auf die Freistellung von Mitarbeiter:innen und der Abstimmung der organisatorischen Abläufe wurden dadurch im Verlauf überwunden.

Aus unserer Sicht stellt das Dilemma zwischen wahrgenommenem Zeitmangel und gleichzeitigem Trainingsbedarf eher eine Führungsaufgabe als eine Machbarkeitsproblematik dar. Studien bestätigen, dass für Kulturwandel und tiefgreifende Änderungsprozesse die Führungsebene unabdingbar ist [28].

Professionelle Identitätsbildung braucht Zeit für Entwicklung und Wachstum [29] sowie kontinuierliches Feedback [30], welche im vorliegenden Konzept durch den Wechsel zwischen gemeinsamen Training im Modul und individuellem Training am Arbeitsplatz über eineinhalb Jahre gewährleistet wurde und durch die fortbestehenden Mentoringgruppen erfolgreich verstetigt wird.

Obwohl mittlerweile klar ist, dass schlechte Kommunikation nicht nur die Patientensicherheit gefährdet [31], sondern die Kliniken auch viel Geld kostet [32, 33], werden medizinische Kommunikationstrainings außerhalb von Studien kaum in der Breite durchgeführt. Mögliche Gründe könnten zum einen sein, dass es im Rahmen von Kommunikationstrainings kaum Daten zu klinik- oder abteilungsindividuellen finanziellen Outcomes gibt und die ökonomische Kosten-Nutzen-Bilanz aus Sicht der Entscheidungsträger:innen schwer überschaubar ist.

Ein weiteres Hemmnis könnte sein, dass Ärzt:innen dazu tendieren, ihre eigenen kommunikativen Fertigkeiten zu überschätzen [34]. Ein mangelndes Bewusstsein für kommunikative Defizite wiederum könnte bei den Entscheidungsträger:innen dazu beitragen, die Wichtigkeit eines Kommunikationstrainings zu unterschätzen.

Im DRG(„diagnosis related groups“)-System ist eine direkte oder indirekte Vergütung von Kommunikationstrainings bisher nicht vorgesehen, wäre prinzipiell aber vorstellbar; bspw. könnten komplexe Gespräche als Leistungsziffer eingeführt werden und an eine Qualifikation in Kommunikation gekoppelt sein.

Zudem konnten wir im KomMent-Projekt feststellen, dass erfahrenere Ärzt:innen ebenfalls wie die teilnehmenden Assistenzärzt:innen von den zur Verstetigung eingeführten Mentoringgruppen profitierten. Die hohe Akzeptanz auf allen Hierarchiestufen führte dazu, dass diese sich auch nach Abschluss des Projekts zur Aufrechterhaltung und Weiterentwicklung der Kommunikationskultur in der Abteilung weiterhin regelmäßig treffen.

Eine Einschränkung der vorliegenden Pilotstudie ist die freiwillige Teilnahme am Training, welche zu einer geringen TN-Zahl führte und möglicherweise eben diejenigen selektierte, welche ohnehin bereits an Kommunikation interessiert waren. Entsprechend wäre für zukünftige Trainings die verbindliche Teilnahme empfehlenswert. Auch für verpflichtende Trainings konnte die Effektivität gezeigt werden [35].

Der Schwerpunkt des Trainings lag entsprechend der Förderung im ärztlichen Bereich. Aufgrund der Bedarfsanalyse wurde das komplette Training auch den Pflegenden angeboten und um zusätzliche interprofessionelle Themen erweitert. Dennoch gaben die Pflegenden in den Abschlussinterviews an, dass der Fokus zu stark auf ärztlichen Bedürfnissen gelegen hätte, auch wenn durch die Integration von Themen, die für funktionierende interprofessionelle Kommunikation und Prozesse wichtig sind, die Teilnahme für Pflegende relevant war. Für Folgetrainings sollten Konzeption und Durchführung von vorneherein interprofessionell ausgelegt werden und stärker die spezifischen Kommunikationsbedürfnisse der Pflegenden aufgegriffen werden.

Obwohl das IKT im Vergleich zu konventionellen Kommunikationstrainings um mehrere wichtige Themen und damit im Stundenumfang erweitert wurde, wurden dennoch Inhalte wie Stärkung der Gesundheitskompetenz der Patient:innen, Einbezug von Selbsthilfegruppen, Stärkung der sektorübergreifenden Zusammenarbeit sowie Förderung der Prävention zu wenig berücksichtigt [22].

Ein modulares IKT für onkologisch tätige Ärzt:innen mit 80 UE, das den Umsetzungsempfehlungen des NKP entspricht, ist in den klinischen Alltag nachhaltig integrierbar. Zukünftig sollten Kommunikationstrainings in der Onkologie interprofessionell, sektorenübergreifend aufgestellt sein und alle Themengebiete von Prävention bis Palliation abdecken sowie die Kompetenz zu transformativem Handeln vermitteln [36].
